# Evolution of a Paradigm Switch in Diagnosis and Treatment of HPV-Driven Head and Neck Cancer—Striking the Balance Between Toxicity and Cure

**DOI:** 10.3389/fphar.2021.753387

**Published:** 2022-01-20

**Authors:** Bouchra Tawk, Jürgen Debus, Amir Abdollahi

**Affiliations:** ^1^ German Cancer Consortium (DKTK) Core Center Heidelberg, German Cancer Research Center (DKFZ), Heidelberg, Germany; ^2^ Clinical Cooperation Units (CCU) Translational Radiation Oncology and Radiation Oncology, National Center for Tumor Diseases (NCT), German Cancer Research Center (DKFZ), Heidelberg University Hospital (UKHD), Heidelberg, Germany; ^3^ Division of Molecular and Translational Radiation Oncology, Heidelberg Ion-Beam Therapy Center (HIT), Heidelberg Faculty of Medicine (MFHD), Heidelberg University Hospital (UKHD), Heidelberg, Germany; ^4^ Heidelberg Institute of Radiation Oncology (HIRO), National Center for Radiation Research in Oncology (NCRO), German Cancer Research Center (DKFZ), Heidelberg University Hospital (UKHD), Heidelberg, Germany

**Keywords:** head and neck (H&N) cancer, human papilloma virus—HPV, radiotherapy, oropharyngeal cancer (OPC), precision medicine, de-intensification trials, patient stratification strategy

## Abstract

More than a decade after the discovery of p16 immunohistochemistry (IHC) as a surrogate for human papilloma virus (HPV)-driven head and neck squamous cell carcinoma (HNSCC), p16-IHC has become a routinely evaluated biomarker to stratify oropharyngeal squamous cell carcinoma (OPSCC) into a molecularly distinct subtype with favorable clinical prognosis. Clinical trials of treatment de-escalation frequently use combinations of biomarkers (p16-IHC, HPV-RNA *in situ* hybridization, and amplification of HPV-DNA by PCR) to further improve molecular stratification. Implementation of these methods into clinical routine may be limited in the case of RNA by the low RNA quality of formalin-fixed paraffin-embedded tissue blocks (FFPE) or in the case of DNA by cross contamination with HPV-DNA and false PCR amplification errors. Advanced technological developments such as investigation of tumor mutational landscape (NGS), liquid-biopsies (LBx and cell-free cfDNA), and other blood-based HPV immunity surrogates (antibodies in serum) may provide novel venues to further improve diagnostic uncertainties. Moreover, the value of HPV/p16-IHC outside the oropharynx in HNSCC patients needs to be clarified. With regards to therapy, postoperative (adjuvant) or definitive (primary) radiochemotherapy constitutes cornerstones for curative treatment of HNSCC. Side effects of chemotherapy such as bone-marrow suppression could lead to radiotherapy interruption and may compromise the therapy outcome. Therefore, reduction of chemotherapy or its replacement with targeted anticancer agents holds the promise to further optimize the toxicity profile of systemic treatment. Modern radiotherapy gradually adapts the dose. Higher doses are administered to the visible tumor bulk and positive lymph nodes, while a lower dose is prescribed to locoregional volumes empirically suspected to be invaded by tumor cells. Further attempts for radiotherapy de-escalation may improve acute toxicities, for example, the rates for dysphagia and feeding tube requirement, or ameliorate late toxicities like tissue scars (fibrosis) or dry mouth. The main objective of current de-intensification trials is therefore to reduce acute and/or late treatment-associated toxicity while preserving the favorable clinical outcomes. Deep molecular characterization of HPV-driven HNSCC and radiotherapy interactions with the tumor immune microenvironment may be instructive for the development of next-generation de-escalation strategies.

## 1 Introduction

Human papilloma virus (HPV)-driven oropharyngeal squamous cell carcinoma (OPSCC) is a subtype of head and neck squamous cell carcinoma (HNSCC) with improved clinical outcomes (Ragin and Taioli, 2007; Ang et al., 2010; Dayyani et al., 2010; Rischin et al., 2010). While the incidence of HNSCCs attributable to tobacco and alcohol (known as “HPV-negative HNSCC”) continues to decrease, the worldwide prevalence of HPV-driven HNSCC has increased to 47.7% since 2005, accounting for ∼73 and ∼72% of oropharyngeal tumors in Europe and the United States (United States), respectively (Mehanna et al., 2013). In the United States, HPV-driven OPSCC has overtaken cervical cancer as the most frequent HPV-driven cancer (Senkomago et al., 2019). HPV type 16, the most prevalent viral driver of carcinogenesis in HPV-driven OPSCC (Dayyani et al., 2010), is the culprit behind 95–100% of this cancer type (Herrero et al., 2003; Ragin et al., 2007; Ragin and Taioli, 2007; Mehanna et al., 2013).

Individuals affected are likely to present at a younger age (less than 60 years) with a history of no or little tobacco consumption and high nodal tumor burden (Fakhry et al., 2008; Ang et al., 2010; Huang et al., 2012; Psyrri et al., 2014). The improved survival outcome has been demonstrated in case series, meta-analyses, and prospective randomized clinical trials (RCTs) (Ragin and Taioli, 2007; Fakhry et al., 2008; Shi et al., 2009; Ang et al., 2010; Dayyani et al., 2010; Rischin et al., 2010; Mehanna et al., 2013), regardless of therapy as long as it conformed to the standard of care (Mirghani et al., 2015a). Similarly, cancer survivorship studies showed a statistically significant difference in survivorship rates between survivors with OPSCC cancers vs. oral cancers (an HPV-negative HNSCC surrogate) (115 individuals per 100,000 per year vs. 16 per 100,000 per year, respectively, *p* < 0.0001) (Patel et al., 2016). In an RCT conducted by the Radiation Therapy Oncology Group (RTOG; RTOG0129), patients with HPV-driven OPSCC had a 58% reduction in the risk of death (HR 0.42, 95% CI 0.27–0.66) and a 51% reduction in risk of disease progression or death (HR 0.49, 95% CI 0.33–0.74) compared to HPV-negative OPSCC (Ang et al., 2010).

To this day, the biological basis of the heightened sensitivity of HPV-driven OPSCC toward treatment is not completely elucidated. To which extent does the interplay between intrinsic properties of the tumor cells vs. the tumor microenvironment affect this radiosensitivity is also an active area of research. Some studies have postulated that expression of wild-type p53 (though inactivated by E6 oncoprotein) persists at low levels and is activated after radiation-induced DNA damage, resulting in cell cycle arrest and death (Kimple et al., 2013). Another study postulated that p16 overexpression leads to an increase in misrepair of DNA double-strand breaks (DSBs) because it inhibits the binding of RAD51, a factor essential for homologous recombination (Dok et al., 2014). This results in a shift toward the non-homologous end-joining pathway (NHEJ) and increased misrepair of DSBs. Cell line experiments have also implicated the cell cycle redistribution of HPV-positive vs. HPV-negative cell lines. HPV + cells lines showed an extensive cell cycle arrest in G2, which could be associated with higher radiosensitivity (Busch et al., 2013; Rieckmann et al., 2013). Additionally, tumor hypoxia is not an inverse prognosticator in HPV + OPSCC(Lassen et al., 2010), although studies have shown no significant difference in tumor hypoxia between HPV + OPSCC and HPV-negative tumors, whether by immunohistochemical staining (Kong et al., 2009), gene signatures (Toustrup et al., 2012), or PET-scans (Mortensen et al., 2012). Finally, the tumor immune microenvironment may play a crucial role in mediating this radiosensitivity. HPV-driven OPSCCs show higher levels of tumor-infiltrating lymphocytes (TILs CD8 T cells) (Balermpas et al., 2016). Radiation therapy causes cellular damage, releasing viral and tumor antigens, which may synergistically activate the immune antitumor response.

The standard of care is based on data from trials conducted irrespective of tumor HPV status, and treatment of advanced stage HNSCC is multimodal par excellence. Non-resectable advanced stage HNSCC is treated with definitive radiochemotherapy (CRT), the standard conventional fractionation scheme being 70 Gray (Gy) in 2 Gy fractions (Fx) with concurrent cisplatin (100 mg/m2) on days 1, 22, and 43 (Pignon et al., 2009). In surgically operable disease, surgery (including reconstruction) is followed by postoperative RT up to 66 Gy (Gregoire et al., 2010). Patients with extracapsular extension (ECE) in the involved lymph nodes (LNs) or positive surgical margins (R) benefit from the addition of cisplatin (100 mg/m2) on days 1, 22, and 43 (Bernier et al., 2005; Gregoire et al., 2010).

**FIGURE 1:**
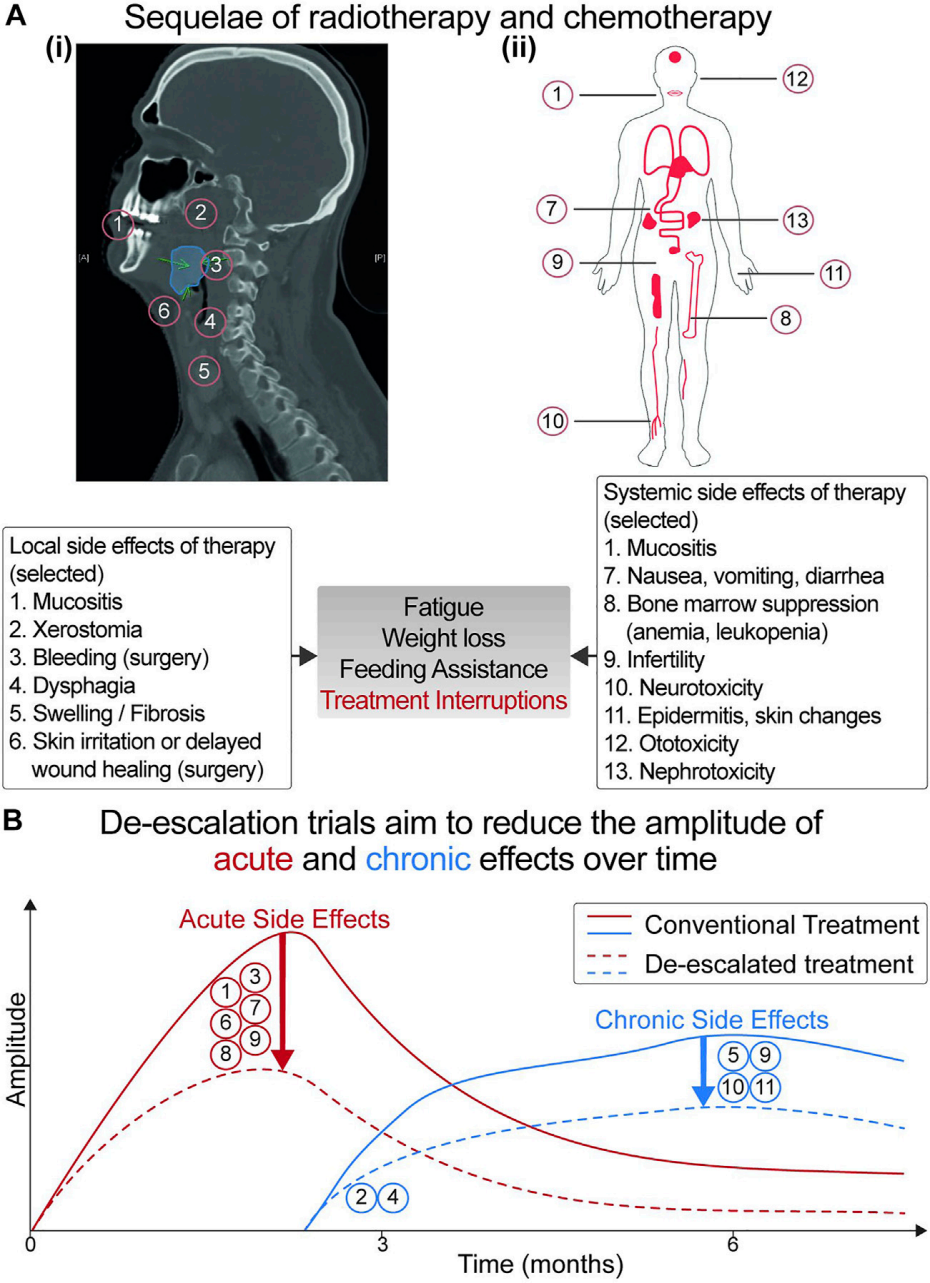
**(A)** Acute and late toxicity profile of local and systemic chemotherapy. **(i)** Sagittal view of a CT-scan shows the patient’s tumor (in blue). Local therapy (surgery and radiotherapy) and systemic treatment can result in acute side effects (occurring within the first 90 days of treatment) or chronic side effects (lasting beyond 90 days). Local side effects include dermatitis, mucositis, xerostomia (dry mouth), dysphagia (difficulty swallowing), bleeding, wound healing swelling, and fibrosis. **(ii)** Systemic side effects are related to the cytotoxic properties of chemotherapy. Increased rates of adverse events (occurring synergistically due to the combination of radiotherapy/chemotherapy) may lead to treatment interruptions, jeopardizing patient outcomes. **(B)** Kinetics of adverse events over time. The aim of de-escalation trials is to flatten the curve of adverse effects [whether acute (in red) or chronic (in blue)], thereby improving the quality of life of patients with HNSCC and cancer survivors.

The toxicity profile accrued per treatment modality (surgery, RT, or chemotherapy) is significant and increases whenever they are combined (summarized in Figure 1A) (Nguyen et al., 2002; Parsons et al., 2002; Pignon et al., 2009; Kelly et al., 2016). Given that patients with HPV-driven OPSCC are younger and will continue to live longer, de-escalation trials were conceived with the aim of decreasing treatment toxicity. Selection of appropriate candidates for treatment de-intensification is crucial to avoid compromising favorable survival outcomes.

This article will briefly discuss the morbidity of treatment modalities in HNSCC. Then, the newest paradigms for diagnosis, risk stratification, and staging of HPV-driven OPSCC will be discussed. Finally, strategic principles behind current de-escalation trials will be summarized, and data emerging from trials that have finished reporting will be discussed.

## 2 Toxicity of Treatment

Toxicity of treatment in HNSCC may be local (to the anatomical region) or systemic as a consequence of cancer burden or administration of chemotherapy. Interruptions or delays in completion of therapy are associated with worsened local control (LC) due to accelerated tumor repopulation (Bese et al., 2007).

Broadly speaking, toxicity can be conceptualized on several domains. Temporally, acute vs. late toxicities are defined as those occurring within 90 days vs. beyond 90 days of treatment completion (Trotti, 2000). Qualitatively, adverse events may be functional or emotional in nature (Trotti, 2000). Quantitatively, the landscape of toxicities (related to surgery, chemotherapy, or RT) can be graded using the Common Terminology Criteria for Adverse Events (CTCAE) (Bentzen and Trotti, 2007). Toxicities are organized according to System Organ Class (SOC) and vary in severity between grade 1 (mild, asymptomatic, and no intervention required), grade 2 (moderate, requiring minimal, local, or non-invasive intervention), grade 3 (severe or medically significant, significantly impairing Activities of Daily Living (ADL), and necessitating hospitalization), and grade 4 (life-threatening and requiring urgent intervention). Grade 5 is death-causing toxicity (Bentzen and Trotti, 2007). Additionally, quality of life questionnaires (QoL) such as the European Organization for Research and Treatment of Cancer—Quality of Life core questionnaire (EORTC-QLQ-C30) or the head and neck–specific module (EORTC-QLQ-HN35) assess the impact of treatment on four domains: psychological, occupational, physical, and social.

As an example of toxicity profiles in the pre–Intensity Modulated Radiotherapy (IMRT) era, in the Intergroup trial, patients randomized to receive RT alone (70 Gy in 2 Gy Fx) had a 51% rate of all grade 3–5 toxicities, the bulk of which was mucositis/dysphagia (32%), followed by dermatitis (13%) and nausea/vomiting (6%). 39% of patients necessitated the use of a feeding tube (Adelstein et al., 2003). Comparatively, in RT with the concurrent cisplatin (100 mg/m2 weekly) arm, grade 3–5 toxicities were significantly increased with an 85% rate of overall toxicities (*p* < 0.0001), 43% mucositis/dysphagia (*p* < 0.08), 40% leukopenia (*p* < 0.001), 18% anemia (*p* < 0.001), 15% rates of nausea/vomiting (*p* < 0.03), and an 8% rate of renal toxicity (*p* < 0.01) (Adelstein et al., 2003).

The patterns of acute symptom burden have been recently described for patients receiving IMRT alone vs. concurrent CRT (Rosenthal et al., 2014). Toxicities were evaluated using the MD Anderson Symptom Inventory—Head and Neck Module (MDASI—HN). For patients receiving IMRT only, in weeks 1–2, the top three most severe symptoms were fatigue, dry mouth, and drowsiness, in decreasing order of severity (Rosenthal et al., 2014). During weeks 6–7, the top three most severe symptoms were problem tasting food, problems with mouth/throat mucus, and difficulty swallowing/chewing. For patients receiving concurrent CRT, there was a statistically significant increase in the overall severity of these symptoms (*p* < 0.001) (Rosenthal et al., 2014).

Most acute side effects usually resolve within months of treatment completion. Conversely, late complications may be milder at onset but can progress over time and be detrimental to the patient’s quality of life (Bentzen and Trotti, 2007). The chronic side effects of RT are dose and volume dependent. Dysphagia rates increase per each 10-Gy increment of the radiation dose to the superior and middle pharyngeal constrictors above 55 Gy (Eisbruch et al., 2004). Risk of aspiration approximates 50% following around 65-Gy dose delivery (Levendag et al., 2007; Feng et al., 2010; Christianen et al., 2015). One-year and 4-year feeding tube dependency rates have been reported to be as high as 41 and 16.7% at 72 Gy, respectively (Garden et al., 2008). Other side effects include tissue fibrosis (Levendag et al., 2007; Feng et al., 2010; Eisbruch et al., 2011), xerostomia, swallowing dysfunction (Langendijk et al., 2009), and development of second primary cancers (Eisbruch et al., 2004, 2011; Nguyen et al., 2006; Machtay et al., 2008; Langendijk et al., 2009; Ramaekers et al., 2011; Mirghani et al., 2015a; Kelly et al., 2016).

With the development of transoral robotic surgery (TORS), surgeons can resect oropharyngeal tumors through the open mouth. According to a case series of 121 patients, 18% of patients experienced Clavien–Dindo grade 3–5 complications (Hay et al., 2017). The most common TORS-related complication was hemorrhage (minor 5.29% vs. major 2.9%) according to a recent meta-analysis (Stokes et al., 2021). Other toxicities included pain at the local site, aspiration-related infections, and dysphagia (Hay et al., 2017). In a case series of 257 patients with HPV-driven OPSCC, post-TORS, moderate and acute dysphagia rates were 14.7 and 8.0%, respectively (Hutcheson et al., 2019). By 3–6 months, moderate–severe dysphagia rates were 0 vs. 13.6% vs. 13.3% in patients treated with TORS alone, TORS + RT, and TORS + CRT, respectively. Gastrotomy tube dependence also increased in patients with increasing treatment intensity. For instance, in a cohort series of 111 patients, addition of adjuvant postoperative RT and CRT increased rates of gastrotomy tube use from 0/13 (0%) to 10/31 (32.3%) and 39/67 (58.2%), respectively (*p* < 0.0002). At 12 months, rates of gastrotomy tubes were 0/13 (0%), 2/31 (6.4%), and 15.9% (10/67) in the TORS alone, TORT + RT, and TORS + CRT groups, respectively, *p* < 0.007 (Sethia et al., 2018).

## 3 Diagnosis and Risk Stratification of HPV-Driven OPSCC

### 3.1 Diagnosis of HPV-Driven Tumors

The first step in managing a patient presenting with a newly diagnosed OPSCC is establishing the presence of an HPV-driven tumor. A crucial distinction must be made between tumors harboring a passenger HPV infection versus those with a transcriptionally active virus. In an HPV-driven tumor, oncoproteins E6 and E7 are transcribed from the virus DNA and expressed in the tumor cells, leading to an interaction with growth regulatory proteins such as tumor suppressors *TP53* (p53) and retinoblastoma (*RB1*), progression into the cell cycle, and acquisition of genomic instability (Münger et al., 2004; Doorbar et al., 2015).

Broadly speaking, there are two classes of HPV testing. Direct tests detect the presence of HPV DNA or RNA, whereas indirect tests establish the presence of HPV *via* molecular surrogates. In clinical settings, the most frequently used direct tests are performed on routine formalin-fixed paraffin-embedded (FFPE) tissue. *In situ* hybridization (ISH) or polymerase chain reaction (PCR) tests detect HPV DNA or RNA (Venuti and Paolini, 2012). Due to the low quality of RNA in FFPE material, detection of HPV E6 and E7 mRNA *via* reverse-transcriptase PCR is infrequently utilized in clinical routines. This method is favored for fresh frozen tissue (Venuti and Paolini, 2012). A promising ISH-based assay (HPV RNAscope) has shown optimal sensitivity and specificity in FFPE tissue but is still not broadly used in clinical practice (Mirghani et al., 2015b).

The most widely used indirect test is p16 immunohistochemistry (p16-IHC), performed on FFPE material (Venuti and Paolini, 2012). Increased expression of p16 (encoded by *CDKN2A* gene) occurs following E7-mediated phospho-RB1 inactivation, allowing p16-expressing tumor cells to bypass cell cycle arrest (Doorbar et al., 2015). The prognostic utility of p16-IHC has been investigated in RCTs (Fakhry and Gillison, 2006; Ang et al., 2010; Rischin et al., 2010; Gillison et al., 2012; Lassen et al., 2013; Fakhry et al., 2014; Masterson et al., 2014), and it is considered an independent prognostic marker for OS in a meta-analysis by the College of American Pathologists (CAP) (Lewis et al., 2018).

However, p16-IHC may represent other physiological or pathophysiological states such as cellular senescence (Rayess et al., 2012). Approximately 10–20% of tumors testing positive for p16-IHC may lack a transcriptionally active HPV infection (Rischin et al., 2010; Singhi and Westra, 2010; Schache et al., 2011; Rietbergen et al., 2013; Rietbergen et al., 2014; Mirghani et al., 2015b; Craig et al., 2019). Patients with p16-IHC+ and HPVDNA-tumors had significantly reduced 5-year OS compared to patients with p16-IHC+ and HPVDNA + tumors (Rietbergen et al., 2013; Rietbergen et al., 2014; Craig et al., 2019) and showed clinical outcomes similar to HPV-negative patients. For instance, in a cohort of 231 patients with OPSCC, 20 patients’ tumors (9%) tested positive for p16-IHC and negative by HPVDNA ISH and HPVRNA PCR (Craig et al., 2019). The 5-year OS in this group was 33 vs. 77% in patients with p16-IHC+ and HPVDNA ISH + tumors (*p* < 0.05) (Craig et al., 2019). A recent meta-analysis compared the performance of standalone p16-IHC, HPVDNA PCR, HPVDNA ISH, and various combinatory testing against the performance of HPVRNA PCR testing (Prigge et al., 2017). The best sensitivity and specificity were achieved with a combination of p16-IHC and HPVDNA PCR testing {sensitivity: 93%, [95% confidence interval (CI) 87–97%], specificity; 96% (95%CI 89–100%) (Prigge et al., 2017)}. The specificity of combining both tests was significantly better than either on its own (*p* < 0.05) (Prigge et al., 2017).

Currently, CAP, the American Joint Committee on Cancer (AJCC), and the Union for International Cancer Control (UICC) eighth-edition TNM staging systems recommend using p16-IHC as a standalone surrogate test for an HPV-driven OPSCC (Lewis et al., 2018). However, the 10–20% false-positive rate of p16-IHC may result in enrolling this patient group into de-escalation trials and undertreating them. Of note, the Eastern Cooperative Oncology Group (ECOG) 1,308 trial, a phase II de-escalation trial of RT based on response to induction chemotherapy (ICT), reported 15/80 (19%) of patients with p16-IHC+ and HPVDNA ISH- tumors (Marur et al., 2017). Compared to patients with p16-IHC+/HPVDNA ISH+, at the 2-year follow-up, these patients had lower PFS rates [0.57 (95%CI 0.28–0.78) vs. 0.83 (95%CI 0.71–0.91)] and OS [0.67 (95%CI 0.05–0.95) vs. 0.98 (95%0.83–0.97)] (Marur et al., 2017). Additionally, within this de-escalated protocol using ICT (Table 1), there were eight local recurrences (LR) and 1 case of distant metastasis (DM) at the 2-year follow-up. These results warrant further follow-up, given previous reports that most treatment failures in HPV-driven OPSCC occur after 2 years of follow-up and are distant metastases in nature (Huang et al., 2013).

**TABLE 1:** Selection of de-escalation trials with reported outcomes: primary chemoradiation: substitution of cisplatin (cis) with cetuximab (cetux).

Study name, ID	AJCC, HPV, smoking	Design and primary endpoint	Adverse events	Survival outcomes
De-ESCALATE NCT01874171 (*n* = 334) Phase III	7th AJCC: T3T4-N0; T1N1-T4N3 8th AJCC:I, II, III HPV testing p16-IHC HPVDNA ISHSmoking<10py	Design: 70 Gy RT + cetuximab vs. cisplatin (100 mg/m^2^). Primary endpoint: overall acute and late severe toxicity	Cetux vs. cis: 2 years number of grade 3–5 events per patient: 4.82 vs. 4.81 (*p* = 0.98)	Cetux vs. cis: 2 years OS: 89.4 vs. 97.5%, *p* = 0.0007 2 year LR: 12 vs. 3%, *p* = 0.0026 2 yearr DM: 9 vs. 3% *p* = 0.0092
RTOG 1016 Phase III, *n* = 987	7th AJCC: T3N0-T4N0; T1T2-N2aN3 8th AJCCI, II, III HPV testing: p16-IHC Smoking<10py	Design: 70 Gy accelerated RT + cetuximab vs cisplatin (100 mg/m^2^). Primary endpoint: 5-year OS (non-inferiority)	Cetuximab vs. cisplatin: no difference in overall rates of acute events (*p* = 0.16); lower mean number of events per patient (2.35 vs. 3.19, *p* < 0.001); no difference in overall rates of late events (*p* = 0.19) or mean number (*p* = 0.12)	Cetux vs. cis: 5 years OS: 77.% vs. 84.6%, *p* = 0.016 5 years LR: 17.3 vs. 9.9%, *p* = 0.0005 5 years DM: 11.7 vs. 8.7%, *p* = 0.09

Most recently, Shinn et al. has retrospectively analyzed the concordance between p16-IHC and HPV-mRNA and its impact on the clinical outcome of 467 patients with oropharyngeal tumors (Shinn et al., 2021). They found a rate of 4.9% discordance between p16-IHC and HPV mRNA (3.4% p16-IHC-/HPV mRNA+ and 1.5% p16 IHC+/HPV mRNA-). Both patient groups had an inferior clinical outcome to double positives. When stratified by HPV mRNA status alone, patients who were p16 negative but HPV mRNA positive had a better outcome than their p16-positive but HPV mRNA–negative counterparts (Shinn et al., 2021).

### 3.2 Not All HPV-Driven OPSCC Are Equal: Risk Stratification in HPV OPSCC.

RCTs (Ang et al., 2010) and large collaborative cohorts (O’Sullivan et al., 2016) identified negative prognostic factors in HPV-related OPSCC.

*Post hoc* analysis of RTOG0129 classified patients with HPV tumors into low or intermediate risk groups based on the N-stage and pack-years of smoking:

• Patients with ≤10py were categorized as the low-risk group regardless of TN staging (Ang et al., 2010).
• Patients with >10py and N0-N2a nodes were also low risk, with 3-year OS rates of 93% (95%CI 88.3–97.7%).
• By contrast, patients with >10py and N2b-N3 tumors were considered intermediate risk with OS rates of 70.8% (95%CI 60.7–80.8%) (Ang et al., 2010).

Additionally, clinical studies revealed that the staging system for HNSCC was not suitable for prognosticating the outcome of HPV-driven tumors as it could not discriminate hazards (Huang et al., 2015; Dahlstrom et al., 2016). In the International Collaboration on Oropharyngeal Cancer Network for Staging (ICON-S), a multi-centric study on 1907 patients in North America, after stratification by the seventh AJCC, patients with Stage I, II, III, and IVa had similar 5-year OS rates of 88, 82, 84, and 81%, respectively. Patients with stage IVb OPSCC had a 5-year OS rate of 60% (O’Sullivan et al., 2016).

Using RPA and adjusted hazard ratios (AHRs), the novel eighth AJCC staging edition was derived (O’Sullivan et al., 2016). In this staging system, p16-IHC is the test for diagnosing an HPV-driven tumor. The T stage remains largely unmodified, and the main consequence is that there are differences between clinical and pathologic N staging, as the N stage was the strongest correlate of OS (Lydiatt et al., 2018). For clinically palpable or radiographically visible disease, the main difference was location of LNs and size (≥6 cm) (Lydiatt et al., 2018). Patients with unilateral LNs smaller than 6 cm are staged cN1 and those with contralateral or bilateral LNs <6 cm are cN2 and any LN ≥ 6 cm confers a cN3 stage (Lydiatt et al., 2018). For surgically resected tumors, the number of LNs (≥5) was the main prognostic factor (Lydiatt et al., 2018). Patients with 1–4 affected LNs and ≥5 LNs were pN1 and pN2, respectively (Lydiatt et al., 2018). ECE was not a prognostic factor in HPV-driven OPSCC and, therefore, is not considered in the updated eighth AJCC staging system (Lydiatt et al., 2018).

This staging system was first developed in patients who received primary CRT and later validated in patients who received surgery followed by adjuvant therapy (Huang et al., 2015; O’Sullivan et al., 2016; Lydiatt et al., 2018).

On this basis, the eighth AJCC staging system for HPV-driven OPSCC was adopted (Lydiatt et al., 2018):

• Stage I: T1-T2 N0-N1 (seventh AJCC equivalent is T1-T2 N0-N2b)
• Stage II: T1-T2 N2 or T3 N0-N2 (seventh AJCC equivalent is T1-2 N2c or T3 N0-N2c)
• Stage III: T4 or N3
• Stage IV: M1

Based on the eighth AJCC, 48% of patients who would have been staged as Stage III or IV according to the seventh AJCC edition migrate to stage I (Lydiatt et al., 2018). Retrospective appraisal of hazard discrimination for the eighth AJCC staging system was conducted in the National Cancer Database (NCDB) for 3,745 patients (Zhan et al., 2017), revealing 4-year OS rates of Stage I (92%), II (81%), and III (63%) (Zhan et al., 2017).

In discussing the eighth AJCC system, it is important to keep in mind that tobacco consumption is not included. Beyond the smoking history, patients with seventh AJCC stage I and stage II OPSCC were candidates for single-modality treatment with excellent outcomes. Patients with stage III–IV 7th AJCC received multimodal therapy (surgery followed by adjuvant CRT or primary CRT). The eighth AJCC staging system was developed based on survival outcomes using retrospectively collected data. The patients with stage III–IVa seventh AJCC who migrated to stage I and II 8th AJCC received more intense therapy compared to patients who were stage I–II in the seventh AJCC and migrated to stage I in the eighth AJCC system.

Consequently, several questions remain to be elucidated. Are the favorable clinical outcomes of these patients related to multimodal therapies reserved for advanced stage OPSCC? Are all stage I HPV OPSCC eligible for treatment de-intensification? Who should receive multimodal therapy? Taken together, several parameters are relevant for evaluation and interpretation of currently completed and ongoing de-escalation trials. First, is the de-escalation arm compared with a “standard of care” arm? What is the primary endpoint? Is the study statistically powered to detect differences in clinical outcomes? Which risk group is this trial targeting (low versus intermediate risk)? How is HPV diagnosis defined? How is the response monitored (clinical/radiographic vs. pathological)? In trials of surgery and adjuvant therapy, what constitutes a negative margin? Finally, questions of cost-effectiveness should be kept in mind when evaluating these trials.

## 4 Principles of De-Escalation Treatment

**FIGURE 2:**
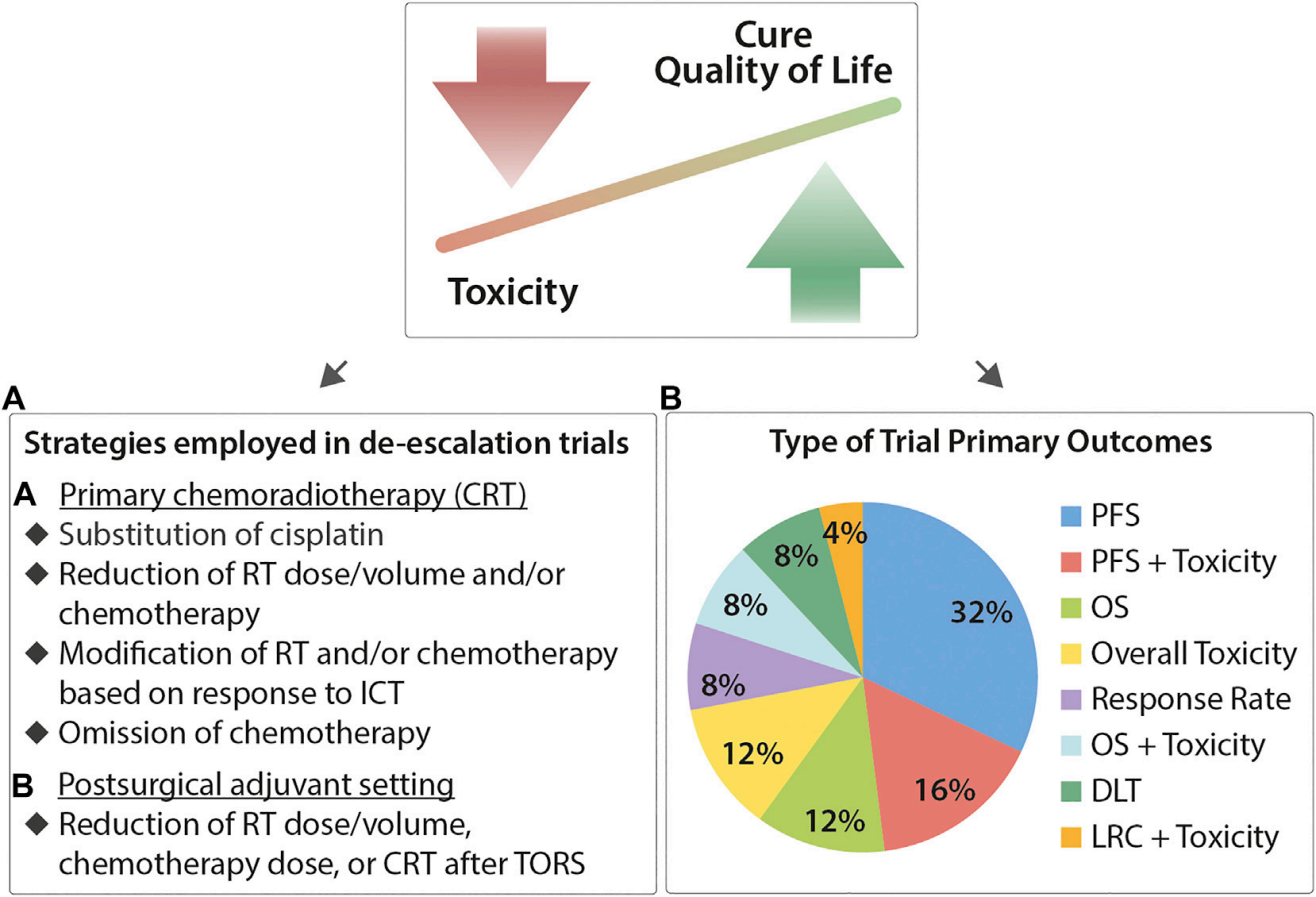
Current principles, de-escalation strategies, and clinical endpoints for therapy de-intensification in HPV-driven OPSCC. The motivation for therapy de-intensification is to decrease toxicity of treatment without compromising patient outcomes. **(A)** In the setting of primary CRT, current strategies focus on dose modification or omission of chemotherapy, substitution of cisplatin (with targeted agents or immunoncology), reduction of radiotherapy dose, or modification of CRT dose based on response to induction chemotherapy. Similar strategies are also employed in the postsurgical adjuvant setting. **(B)** Survey of primary clinical endpoints under investigation in de-escalation trials. Not all trials are powered to investigate changes in the clinical outcome. (DLT, dose-liming toxicity; RR, response rate.)

The overarching aim is the identification of appropriate treatment intensity that minimizes morbidity of cancer survivors without compromising their survival prospects, as seen in Figures 1B, 2 below.

De-escalation trials follow one or a combination of the following strategies. In the primary RT/CRT treatment setting, strategies followed include the following:

• Reduction of chemotherapy toxicity by replacing cisplatin with targeted agents (e.g., anti-EGFR treatment with cetuximab)
• Reduction of chemotherapy and or RT dose/volume
• Omission or modification of chemotherapy dose or RT dose/volume depending on clinical or pathologic response to ICT
• Omission of chemotherapy

In the surgical and adjuvant treatment setting, the strategy includes reduction or omission of RT, chemotherapy, or CRT after surgery. Additionally, emerging clinical trials are evaluating the combination of immunotherapy with radiotherapy (sequential or concomitant) (NCT02764593, 2016; Spreafico et al., 2018) and the use of particle therapy with protons instead of conventional photon radiotherapy to reduce toxicity to the surrounding tissue (Gunn et al., 2016).

### 4.1 De-Escalation Trials in Primary (Chemo)Radiotherapy

#### 4.1.1 Combining Radiotherapy With Cetuximab

Cetuximab is a monoclonal antibody targeting the epidermal growth factor receptor (EGFR), which mediates the activation of oncogenic pathways in HNSCC. In 2006, the Bonner RCT prospectively evaluated the impact of adding cetuximab to RT in patients with advanced-stage HNSCC (Bonner et al., 2006). Compared to patients who received RT alone, there was a statistically significant survival advantage without a concomitant increase in radiation-induced toxicity (median OS 29.3 vs. 40 months, respectively) (Bonner et al., 2006). The survival advantage was strongest among patients with clinical features suggestive of HPV-driven HNSCC, namely, young patients with oropharyngeal tumors, smaller primaries, and higher nodal involvement (Bonner et al., 2006), a finding subsequently confirmed upon secondary analysis based on p16-IHC status (Rosenthal et al., 2016).

At the present date, three prospective clinical trials, RTOG1016, De-ESCALaTE HPV, and TROG12.01, have evaluated the impact of adding cetuximab to primary RT of 70 Gy. In these trials, non-inferiority of cetuximab was not achieved and cisplatin-based CRT consequently remained the standard of care in HPV-driven OPSCC treated with primary RT (Gillison et al., 2019; Mehanna et al., 2019). The findings are summarized in Table 1 below.

In the De-ESCALaTE HPV trial (NCT01874171), 304 patients with T3T4-N0 and T1T4-N1N3 (seventh AJCC) p16-IHC + OPSCC and <10py smoking received 70 Gy RT with cisplatin (100 mg/m2 every 3 weeks) or cetuximab. The primary endpoint was overall (acute and late) grade 3–5 toxicities. The study was powered to detect a 25% reduction in overall toxicities in the cetuximab arm. No significant differences in the overall mean number of grade 3–5 toxicity events per patient (cetuximab 4.82 vs. cisplatin 4.81, *p* = 0.98), acute severe toxicities (both arms scored 4.4, *p* = 0.84), and severe late toxicities (cetuximab: 0.5 vs. cisplatin 0.4, *p* = 0.53) were detected. Similarly, there was no difference measured in the global QoL score at 2 years (*p* = 0.99), as measured by EORTC-QLQ-C30. Additionally, there was no difference in QoL scores or dysphagia (as measured by the MD Anderson Dysphagia Index [MDADI]). Nevertheless, a lower number of serious adverse events (SAE) were observed in the cetuximab arm (95 vs. 162 with cisplatin, *p* < 0.0001). Finally, at the 2-year follow-up, OS was significantly inferior in the cetuximab arm vs. the cisplatin arm (89.4 vs. 97.5%, *p* = 0.0007), with increased rates of LR (12 vs 3%, *p* = 0.0026) and DM (9 vs 3%, *p* = 0.0092).

RTOG1016 (NCT01302834) was a non-inferiority RCT randomizing 987 patients with p16-IHC + OPSCC to cisplatin (100mg/m2 every 3 weeks) or cetuximab with accelerated RT (70Gy in 35 fx, six fx per week). The primary endpoint was the 5-year OS. The trial revealed worsened rates of OS in the cetuximab arm [77.9% (95%CI 73.4–82.5) vs. 84.6% (95%CI 80.6–88.6), *p* = 0.016], worsened PFS (67.3 vs. 78.5%, *p* = 0.0002), increased rates of LR (17.3 vs. 9.9%, *p* = 0.0005), and a non-significant trend toward increased DM (11.7 vs. 8.6%, *p* = 0.09) (Gillison et al., 2019). Consequently, non-inferiority of cetuximab was not achieved and cisplatin-based CRT remains the standard of care in HPV-driven OPSCC treated with primary RT (Gillison et al., 2019; Mehanna et al., 2019). The findings are summarized in Table 1 below.

A third trial, TROG12.01 (NCT01855451), randomized 182 patients with p16-IHC + OPSCC (seventh AJCC) (T1T2-N2c or T3-N0N2c) to receive RT 70 Gy in 35Fx with cisplatin weekly (dose-reduced, 40 mg/m2) or cetuximab (Rischin et al., 2021). The primary endpoint was toxicity (acute and at 2 years), measured by the MDADI and MDASI-HN. There was no difference in toxicities between both arms (Rischin et al., 2021). However, there was a significant decrease in the 3-year failure-free survival rates in the cetuximab arm (80%) versus the cisplatin arm (93%) [HR = 3.0 (95% CI: 1.2–7.7); *p* = 0.015] (Rischin et al., 2021).

For all de-escalation trials, the biomarkers used for selection of HPV-driven tumors, the AJCC staging (seventh and corresponding eighth when applicable), and the smoking status of patients enrolled will be described in the adjacent tables.

#### 4.1.2 Reduction of Radiotherapy/Chemoradiotherapy Dose

NCT01530997 is a phase II trial, where patients with tumor stages T0-T3, N0-2c, and M0 (seventh AJCC) and <10py smoking history were treated with 60 Gy IMRT over a 6-week period with concurrent dose-reduced weekly cisplatin (dose-reduced, 30 mg/m2). The primary endpoint was pathologic complete response (pCR). Toxicity was measured using physician-reported outcomes and Patient-Reported Outcomes CTCAE (PRO-CTCAE). QoL was evaluated using the EORTC-QLQ-HN35. At a 10-Gy total dose reduction, the pathologic complete remission (pCR) rate was 86% (37/43). The 2-year OS, LC, disease-free survival (DFS), and PFS were 95, 100, 100, and 100%, respectively (Chera et al., 2017). 15/43 (39%) of patients required a feeding tube for a median of up to 15 weeks following treatment, but none permanently. Moreover, there were no grade 3 late adverse effects (Chera et al., 2017).

Consequently, a larger trial with 114 patients (NCT02281955) was planned with 2-year PFS as the primary endpoint (Chera et al., 2019). The study was powered to detect a 2-year PFS of 87% or greater, with the alternate hypothesis that the PFS was 80% or less. Patients with eighth AJCC stage I tumors (T1–T2 and N0–N1) received standalone RT (60 Gy/2 Gy Fx), and patients with stage II–III tumors received 60 Gy RT with concurrent weekly cisplatin (30 mg/m^2^) (Chera et al., 2019). Clinical response was assessed using positron emitted tomography (PET) and computed tomography (CT) imaging at 10–16 weeks, omitting post-treatment biopsies and selective neck dissection (Chera et al., 2019). The PET/CT complete response rate was 93 and 80% at the primary tumor site and the neck, respectively (Chera et al., 2019). The 2-year PFS and OS were 86 and 97%, respectively. 34% of patients required feeding tubes acutely, with none developing feeding tube dependence (Chera et al., 2019). There were no grade 3 or higher late adverse events reported (Chera et al., 2019). Mouth dryness was the greatest symptom burden, with no return of function to the baseline after 1 year (Chera et al., 2019).

NRG-HN002 is another trial where 316 patients, classified as Ang low risk (Ang et al., 2010) (i.e., seventh AJCC T1T2-N1N2b or T3-N0N2b, <10py), were randomized to either IMRT (60 Gy/2 Gy Fx) with concomitant weekly cisplatin (40 mg/m2) or accelerated standalone IMRT 60 Gy in 5 weeks (Yom et al., 2021). For either arm to progress into a phase III trial, the co-primary endpoint was a 2-year PFS rate more than the historic control of 85% and an acceptable dysphagia toxicity measured by an MDADI score ≥60. Patients in the IMRT + Cisplatin arm had a PFS of 90.5% (*p* = 0.035) and an acceptable 1-year MDADI mean score of 85.3% (Yom et al., 2021). By contrast, the IMRT alone arm failed to meet the acceptability criterion for non-inferiority with a PFS of 87.6% (*p* = 0.228). The 1-year MDADI mean score was adequate with 81.76%. Strikingly, patients in the IMRT alone arm had a higher rate of locoregional failures (LRF: 9.5 vs. 3.3%) [HR = 0.39 (95% CI, 0.17 to 0.90), *p* = 0.02], with most failures occurring at the primary tumor site (Yom et al., 2021). Toxicities were higher for the IMRT + Cisplatin arm than the IMRT alone arm, but 2-year late toxicities were comparable (grade 4: 1.3 vs. 1.4%, grade 3: 20.0 vs. 16.7%) (Yom et al., 2021). The 2-year OS was 96.7% in the IMRT + Cisplatin arm and 97.3% in the IMRT alone arm (Yom et al., 2021). The trial has currently advanced to phase III, where de-intensified IMRT [60 Gy/2GFx) + weekly Cisplatin (40 mg/m2)], de-intensified IMRT (60 Gy/2Fx) + nivolumab, and 70 Gy IMRT + weekly Cisplatin (40 mg/m2) will be directly compared. The co-primary endpoints are PFS and the MDADI QoL score (Yom et al., 2021).

Data from these trials (summarized in Table 2 below) are in agreement with De-ESCALaTE HPV and RTOG1016 regarding the importance of concurrent cisplatin in primary CRT. Nevertheless, with a 10Gy reduction in the RT dose and 20–40% reductions of the cisplatin dose (from 300 mg/m2 to 180–240 mg/m2), clinical and functional outcomes were encouraging. The main limitation is the short follow-up duration, given that distant metastases are detected in this patient population from 2 years on after treatment (Huang et al., 2013).

**TABLE 2:** Selection of de-escalation trials with reported outcomes: primary chemoradiation: de-escalation of chemoradiotherapy dose.

Study name, ID	AJCC, HPV, smoking	Design and primary endpoint	Adverse events	Survival data
NCT01530997 *n* = 43	7th AJCC T0T3-N0N2c 8th AJCC I, II, III HPV testing: p16 IHC or HPV ISH Smoking:<10py	Design: Stage I: RT 60 Gy. All others: 60 Gy RT + weekly cisplatin (30 mg/m2). Response monitoring: pathologic. Primary endpoint: pathologic complete response	Feeding tube: during treatment: 39%, 0% permanent; EORTC QLQ QLO–C30: pre and 2 years post global 80/82 (lower worse); CTCAE: 0% grade 3–4 adverse events at 36 months	pCR: 86% 2 years OS: 95% 2 yearS PFS: 100% 2 years: LC: 100% 2 years DM: 100%
NCT02281955 *n* = 114	7th AJCC: T0T3 N0N2c 8th AJCC I, II, III HPV testing: p16 IHC or HPV ISH Smoking: 80% with <10 py 20%with >10 py	Design: Stage I: RT 60 Gy. All others: 60 Gy RT + weekly cisplatin (30 mg/m2). Response monitoring: post-treatment PET and CT. Primary endpoint: 2-year PFS	Feeding tube during treatment: 34%, 0% permanent; EORTC QLQ QOL–C30: pre and 2 years post global 79/84 (lower worse); CTCAE: 0% grade 3–4 adverse events at 36 months	2 years PFS: 86% 2 years OS: 95% 2 years LR: 95% 2 years DM-free survival (DMFS): 91%
NRG HN002 NCT02254278 *n* = 316	7th AJCC: T1T2-N1N2b T3-N0N2b 8th AJCC I, II HPV testing: p16 IHC Smoking:<10py	Design: Arm 1: IMRT 60 Gy in 6 weeks + cisplatin (40 mg/m2). Arm B: IMRT alone 60 Gy in 5 weeks. Primary endpoint: 2-year PFS acceptability >85% with an MDADI threshold of >60% (*α* = 0.05)	IMRT + Cis vs. IMRT 1 year MDADI 85.3 vs. 81.76%; CTCAE: acute toxicity; Grade 4: 15.1 vs. 2.0%; Grade 3: 65.5 vs. 50.3%. Late toxicity: Grade 4: 1.3 vs. 1.4%; Grade 3: 20.0 vs. 16.7%	IMRT + Cis vs IMRT alone: 2 years PFS: 90.5 vs. 87.6% IMRT arm did not meet acceptability criterion (>85%, *p* = 0.228) 2 years OS: 96.7 vs. 97.3% 2 years LRF: 3.3 vs 9.5% (*p* = 0.02)

#### 4.1.3 Modulation of Treatment According to Response to Induction Chemotherapy (ICT)

Historically, response to cisplatin-based ICT was considered a good predictor of radiation sensitivity (Mirghani et al., 2015a). The first trial exploring ICT in HPV-driven OPSCC was ECOG 2399 (Fakhry et al., 2008)^79^, whereby patients with oropharyngeal or laryngeal tumors (seventh AJCC T2-N1N3 or T3T4-N0N3) received two cycles of induction, paclitaxel and carboplatin, followed by CRT (70Gy RT with paclitaxel) (Fakhry et al., 2008). The primary endpoint was organ preservation, defined as freedom from primary site salvage surgery or primary tumor recurrence. For the subset of patients with HPV-driven OPSCC, 2-year OS and PFS were 95% and 86%, respectively (Fakhry et al., 2008). Nevertheless, high toxicity rates were observed, with 54–53% grade 3 or worse rates of dysphagia and mucositis (Cmelak et al., 2007). 26% of patients required gastrotomy tube placement during treatment, and 17% were dependent on tube feedings at 6 months (Cmelak et al., 2007).

Therefore, ICT-based de-escalation trials utilize the principle of monitoring tumor response after ICT to guide the decision toward a decrease in RT or CRT doses (selected trials in Table 3). In ECOG 1308 (NCT01084083), patients with resecteable OPSCC (seventh AJCC T3-T4b, N0-N3) received three cycles of ICT with cisplatin, paclitaxel, and cetuximab (Marur et al., 2017). Their next treatment was selected based on their clinical response to ICT. Patients with clinical complete response (CR was assessed by clinical examination using endoscopy and CT or magnetic resonance imaging (MRI)) received de-escalated RT 54Gy with concurrent cetuximab. Partial responders received 69.3 Gy with concurrent cetuximab (Marur et al., 2017). The 2-year OS and 2-year PFS were 94% (95%CI 82–98) and 80% (95%CI 65–89) for patients who achieved a primary site CR and were treated with 54 Gy of radiation. For all evaluated patients, the 2-year OS and PFS rates were 91% (95%CI 82–96) and 78% (95% CI 67–86), respectively (Marur et al., 2017). Additionally, this trial reported significantly lower rates of difficulties swallowing solids in patients receiving 54 vs. 69 Gy (40 vs. 89%, *p* = 0.01) and impaired nutrition (10 vs. 44%, *p* = 0.025), as measured by the Vanderbilt Head and Neck Symptom Survey-version 2 (VHNSSv2) (Marur et al., 2017). Nonetheless, 13/80 patients (16%) had strong protocol deviations in this trial (Marur et al., 2017), and several patients had dose reduction of cisplatin (17.5%), cetuximab (22.5%), and carboplatin (2.5%), respectively, due to grade 3 or more toxicity (CTCAE) during induction, raising the question of whether addition of ICT-associated toxicity for patient selection should not be considered to assess the net benefit of treatment de-escalation (Marur et al., 2017; Mirghani and Blanchard, 2018; Wirth et al., 2019). Finally, a *post hoc* analysis of this trial suggested worsened outcomes for patients with >10 py of smoking (Marur et al., 2017).

**TABLE 3:** Modulation of radiotherapy or chemoradiotherapy dose according to response to induction chemotherapy (ICT).

Study name, ID	AJCC, HPV, smoking	Design and primary endpoint	Adverse events	Survival data
ECOG 1308 Marur et al. (2017) NCT01084083 *N* = 80	7th AJCC: T3T4N0, T1N1-T4N3 8th AJCC: I, II, III HPV testing: p16 IHC or HPV ISH Smoking: 39% pts >10py	Design: ICT: 3 cycles of cisplatin, paclitaxel, and cetuximab; then cCR: RT 54Gy + cetuximab; no cCR: 69.3 Gy + cetuximab. Response monitoring: clinical. Primary endpoint: 2-year PFS (powered to expect 85% in patients with cCR after induction and 54 Gy)	54 vs. 69.3 Gy: 1 year swallowing dysfunction: (40 vs. 89%, *p* = 0.011); 1 year impaired nutrition (10 vs. 44%, *p* = 0.025); 18/80 (22.5%) patients with ICT protocol deviation	cCR group treated with 54 Gy, (*n* = 51): 2 years PFS 80%, 2 years OS 94% No cCR with 69 Gy (*n* = 15): 2 years PFS 67%, 2 years OS 87% p16IHC+and HPVISH+: 2 years PFS HR = 0.83, OS HR = 0.93 p16IHC + but HPVISH-2 years PFS = 0.57, OS 0.87
CCRO-022 Chen et al. (2017) NCT01716195 NCT02048020 N = 45	7th AJCC: III-IV 8th AJCC: I-II-III HPV testing: p16 IHC Smoking: 24.4% > 10py	Design: 2 cycles of ICT paclitaxel–carboplatin; then, responders: RT 54 Gy + weekly paclitaxel. Non-responders: RT 60 Gy + weekly paclitaxel. Response: clinical radiography. Primary endpoint: 2-year PFS (72 vs. 86% as thresholds for inefficacy vs. efficacy of trial *α* = 0.09)	FACT- H&N During ICT: 39% grade III adverse events including 39% leukopenia. During CRT: grade III dysphagia (20%) 2 years grade III + mucosal–esophageal toxicity: no difference in 54 vs. 60 Gy (*p* = 0.47)	2 years PFS: 92% 2 years OS: 98% 2 years LR: 95% 2 years DM: 98%
OPTIMA NCT02258659 *N* = 62	7th AJCC: T1T4-N2N3 T3T4-anyN 8th AJCC: I-II-III stratified into: low risk *n* = 28, ≤T3 and ≤N2b and ≤10py High Risk n = 34, T4 or ≥ N2c or >10py HPV testing: p16-IHC or HPVDNA PCR or HPVRNA ISH Smoking: 35% > 10py	Design: ICT with nab-paclitaxel + carboplatin; then, low risk + >50% pCR after ICT: 50 Gy RT low risk + 30–50% pCR OR high risk + >50% pCR: CRT 45 Gy (THFX). All others: CRT 75 Gy (THFX) Response monitoring: pathologic response. Primary endpoint: 2-year PFS to detect non-inferiority to historical control (85%)	Toxicities (CTCAE) for RT50 < CRT45 < CRT75; acute grade III + mucositis (30, 63, 91%, *p* = 0.004); acute grade III + dermatitis (0, 20, 55%, *p* < 0.00001); PEG-tube requirement (0, 31,82%, *p* < 0.001)	Non-inferiority demonstrated: 2 years overall PFS: 94.5% 2 years PFS: 100% in low risk, 92% for high risk 2 years OS: 100% low risk, 97% in high risk 2 years LC: 100% low risk, 97% in high risk 2 years DM: 100% low risk, 100% high risk
Quarterback trial NCT01706939 (*n* = 23) Misiukiewicz et al. (2019)	7th AJCC: T3T4-N0 T1N1-T4N3 OPSCC, Nasopharynx or CUP 8th AJCC: I-II-III HPV testing: p16-IHC and HPVDNA PCR Smoking: <20 py	Design: ICT TPF followed by complete or partial remission randomized to - RT 56 Gy + weekly carboplatin - RT 70 Gy + weekly carboplatin. None responders: RT70 Gy + weekly carboplatin. Primary endpoint: 3-year non-inferior PFS, LC	Trial ended early	56 vs. 70 Gy 3 years PFS 83.3 vs. 87.5% 3 years OS: 83.3 vs. 87.5%. Non-inferiority could not be determined

The Optima non-inferiority trial stratified 62 patients with oropharyngeal tumors based on risk factors (low risk: seventh AJCC ≤ T3 ≤N2b ≤ 10py, high risk: T4 or ≥ N2c or >10py) and pathological response to three cycles of ICT with nab-paclitaxel and carboplatin (≥50% response vs. 30 to <50% response vs. <30%) (Seiwert et al., 2019). Low-risk patients with ≥50% response after ICT received standalone RT (50Gy in 2Gy over 5 weeks) (Seiwert et al., 2019). Low-risk patients with 30 to <50% response and high-risk patients with ≥50% response received CRT 45 Gy in 1.5 Gy Fx twice daily with concurrent paclitaxel, 5-Fluorouracil, and hydroxyurea (THFX) (Seiwert et al., 2019). All other patients (low risk with <30% response and high risk with <50% response) received CRT 75 Gy in 1.5 Gy Fx twice daily and concurrent THFX (Seiwert et al., 2019). The primary endpoint was 2-year overall PFS, with the study powered to allow 11% difference from the historical control of 85%. At 2 years, PFS was 94.5%, proving non-inferiority. There were 28 low-risk and 34 high-risk patients in this trial, respectively (Seiwert et al., 2019). Toxicities increased significantly as regiments increased in intensity (RT50Gy < CRT45Gy < CRT75Gy) with acute grade 3–4 mucositis rates of 30, 63, and 91%, respectively (*p* = 0.004), and gastrotomy tube requirements of 0, 20, and 55%, respectively (*p* = 0.004) (Seiwert et al., 2019). 82% of patients received de-escalated treatment (RT50Gy or CRT45Gy), with 2-year PFS being 100% in the low-risk group and 92% in the high-risk group (Seiwert et al., 2019). In a similar vein, 2-year OS was 100% in the low-risk group and 97% in the high-risk group (Seiwert et al., 2019).

The Quarterback trial was a planned prospective randomized control trial, where patients received three cycles of induction with docetaxel, cisplatin, and 5-Fluorouracil (TPF) (Misiukiewicz et al., 2019). Complete or partial responders (as monitored by PET-CT or biopsies) would be randomized to 56 Gy IMRT or 70 Gy IMRT with weekly carboplatin (Misiukiewicz et al., 2019). Non-responders would receive the standard 70 Gy CRT arm (Misiukiewicz et al., 2019). The primary endpoint was non-inferiority with 3-year PFS. The trial closed early with 23 patients enrolled (Misiukiewicz et al., 2019). Although 20 patients developed significant response to ICT and were randomized, non-inferiority could not be demonstrated (*p* = 0.8) (Misiukiewicz et al., 2019).

### 4.2 De-Escalation of Post-Surgical Treatment

#### 4.2.1 De-Escalation of Adjuvant Radiochemotherapy

Stratification of patients after surgery based on their pathological results aims to identify patients who can benefit from the complete omission of postoperative radiation and chemotherapy (Kelly et al., 2016), (see Table 4).

**TABLE 4:** Surgical approaches: de-escalation of adjuvant radiotherapy or chemoradiotherapy.

Study name, ID	AJCC, HPV, smoking	Design and primary endpoint	Adverse events	Survival data
MC1273 Ma et al. (2019) NCT01932697 *N* = 80	7th AJCC: III-IV with high risk features: ECE or LVI, PNI ≥2 LN, any LN > 3 cm or ≥ T3) 8th AJCC: I-II-III HPV testing: p16-IHC Smoking: <10 py	Design: surgery (R0) + neck dissection. Cohort A: ECE-: 30 Gy/1.5 Gy twice daily + 15 mg/m2 docetaxel. Cohort B: ECE+: 36 Gy/1.8 Gy twice daily + docetaxel. Primary endpoint: 2-year LRC rate of 20% or less (with 2-sided 85% CI) and <20% rate of acute grade 3 or worse toxicity, *α* = 0.06	2-year grade III toxicity (CTCAE): 0%	2 years LC: cohort A; 100%, cohort B: 93% 2 years DM: cohort A: 97.2%, cohort B 79% 2 years PFS: 91.1% 2 years OS: 98.7%
NCT02760667 Sadeghi et al. (2019) *n* = 54	7th AJCC: T1T2-N1N2cT3-N0N2c T4-N0N2c 8th AJCC: I-II-III HPV testing: p16-IHC Smoking: Unknown	Design: 3 cycles of ICT (cisplatin + docetaxel) and then TORS + ND. Primary endpoint: pathologic response	—	Complete pathologic response: primary tumor: 72%; nodal site: 57%; both: 44%
ECOG3311 NCT01898494 *N* = 511 Ferris et al. (2020)	7th AJCC: T1T2-N1N2b 8th AJCC: I-II HPV testing: p16-IHC Smoking: Unknown	Design: low risk: Arm A: TORS only; intermediate risk (R0, N2, ECE<1 mm): Arm B: TORS +50 Gy IMRT Arm C: TORS + 60 Gy IMRT; high risk (R1, ECE+) into Arm D: TORS + 66 Gy IMRT + cisplatin (40 mg/m2). Primary endpoint: 2-year PFS, grade 3–4 bleeding events during surgery, and positive margins	—	2-year PFS: Arm A: 96.9%; Arm B: 94.9%; Arm C: 96.0%; Arm D: 90.7%
DART-HPV NCT02908477 *N* = 194	7th AJCC: ≥T3, ≥N2, LVI, PNI and R0 HPV testing: p16-IHC 8th AJCC: II-III Smoking: <10py	Design: TORS and then intermediate risk: ECE-Twice daily RT30 Gy/1.5 Gy + Docetaxel; high risk: ECE + Twice daily RT36 Gy/1.8 Gy + Docetaxel. Standard arm: RT 60 + cisplatin weekly (40 mg/m2)	2-year grade III AES (CTCAE): 1.6% for the experimental arm and 7.1% for the standard *p* = 0.058	2 years PFS 86.9 vs. 95.8% for experimental vs. standard pN2 and ECE: 2 years PFS 42.9% for experimental arm vs. standard

ECOG3311 is a phase II trial where 445 patients with intermediate risk OPSCC (seventh AJCC T1T2-N1N2b p16-IHC + OPSCC) were randomized into four clinical arms based on the presence/absence of pathological risk factors after TORS resection of the primary tumor and neck dissection (NCT01898494, 2013). Patients with 0-1 LNs, no ECE, and negative margins did not receive subsequent adjuvant treatment (arm A) (Ferris et al., 2021). Patients with R0, N2 disease, or ECE <1 mm received de-escalated RT (in one of two possible arms: Arm B 50 Gy or Arm C 60 Gy). Arm D consisted of patients with R1, >4 involved LNs, or ECE who received CRT (66Gy RT + weekly cisplatin 40mg/m2) (Ferris et al., 2020; Ferris et al., 2021) Co-primary outcomes were 2-year PFS>85%, accrual rate, grade 3–4 bleeding events during surgery, and positive resection margins (Ferris et al., 2020). The positive margin rate was 3.3 and 5.9% grade III or IV oropharyngeal bleeding (Ferris et al., 2021). This trial also met its primary endpoint for PFS: 2-year PFS for Arms A, B, C, and D were 96.9, 94.9, 96, and 90.7%, respectively (Ferris et al., 2021).

MC1273 is a trial which enrolled patients with intermediate-risk HPV-driven OPSCC and R0 surgeries to receive de-escalated adjuvant CRT (Ma et al., 2019). Intermediate risk criteria were defined as seventh AJCC stage III–IV and high-risk features such as ECE, lymphovascular invasion (LVI) or perineural invasion (PNI), ≥2LN, any LN > 3 cm, or ≥ T3 (Ma et al., 2019). Patients with >10py history were excluded (Ma et al., 2019). ECE was the stratifying factor whereby patients with no ECE (cohort A) received 30 Gy RT in 1.5 Gy twice daily fractions and concurrent docetaxel (Ma et al., 2019). Cohort B consisted of patients with ECE, who received 36 Gy in 1.8 Gy twice daily fractions and concurrent docetaxel (Ma et al., 2019). The primary endpoint was 2-year LC with rates of 100 and 93% in cohorts A and B, respectively (Ma et al., 2019). 2-year PFS and OS for all patients were 91.1 and 98.7%, respectively (Ma et al., 2019). No patient required a gastrotomy tube by 1 month after treatment (Ma et al., 2019). A subsequent phase III trial, DART-HPV, has been designed, where patients were randomized to RT (twice daily, 30 Gy/1.5 Gy or 36/Gy in 1.8 Gy with concomitant docetaxel) or RT 60Gy/2Gy once daily and cisplatin weekly (40 mg/m2) (NCT02908477, 2016; Ma et al., 2019) The primary endpoint was grade 3 AE preliminary results that were presented at the annual 2021 American Society of Radiation Oncology (ASTRO) meeting. Grade 3 AEs were 1.6% for the experimental arm vs. 7.1% for the standard of care (*p* = 0.058). However, 2-year PFS was 86.9 vs. 95.8%. Particularly, patients with pN2 disease and ECE had the worst outcomes after de-escalation, with 42.9% PFS rates compared to 100% in the standard of care arm.

Taken together, the outcomes of ECOG3311 and MC1675 provide an encouraging basis for de-escalation of therapy in the adjuvant setting. However, patients with ECE + pN2 disease may not be suitable for treatment de-escalation. In MC167, the 2-year PFS was 42.9% for these patients who were in the de-escalation arm of the trial, with 77% LRC and 59.4% DMFS rates. By contrast, those patients had a 100% 2-year PFS in the standard of care arm. Similar results were seen in ECOG3311, whereby patients with either ECE or >4LNs received a standard dose CRT (66 Gy) with weekly cisplatin and had a 2-year PFS of 90.7%. This once again highlights the importance of adequate patient selection.

Further data are also awaited from two prospective trials, PATHOS and DELPHI. The PATHOS trial stratifies 242 patients with OPSCC seventh AJCC T1T3-N0N2b disease into four arms as well, depending on pathological results after TORS (Owadally et al., 2015). Patients with no risk factors go into an observational alone arm, patients with intermediate risk factors (seventh AJCC T3 stage, pN2a-N2b, LVI, pNI, or close margins) receive RT only (50 Gy or 60 Gy), high-risk patients with R1 resections receive 60 Gy RT, and patients with ECE receive CRT 60 Gy with cisplatin (100 mg/m2) (Owadally et al., 2015). Co-primary outcomes are 1-year MDADI and 1-year OS (Owadally et al., 2015).

The ongoing DELPHI trial (NCT03396718, 2018) aims to enroll 384 patients into two clinical arms of radiation dose de-escalation based on pathological risk factors. In the first level of the DELPHI trial, patients with intermediate risk (pT3, R0 margins, ≤ involved LNs, and no ECE) receive a 10% reduction of standalone RT (54 Gy to the tumor bed) and 45 Gy to the cervical LNs. Patients with at least one high-risk feature (R1 status, pT4, ≥4LNs, or ECE) will receive 59.4 Gy for the tumor bed, 45Gy to the cervical LNs, and additional chemotherapy. The primary endpoint is 2-year locoregional recurrence. If no more than three tumor recurrences in 30 patients occur in the first 2 years, further de-escalation of the RT dose will ensue, whereby patients with no high-risk features will receive 48.4 and 39.6 Gy to the tumor bed and cervical LNs, respectively. Patients with high-risk features will receive 55 and 39.6 Gy to the tumor bed and cervical LNs, respectively (NCT03396718, 2018).

The ADEPT trial (NCT01687413, 2012) was comparing 60 Gy RT alone vs. 60 Gy RT + weekly cisplatin 40 mg/m2 in stage I–III HPV-driven OPSCC with ECE. However, this trial has terminated due to slow accrual.

### 4.3 Comparison of Primary Radio(Chemotherapy) Versus Postoperative Adjuvant Radio(Chemo)Therapy

Between 2004 and 2013, the percentage of patients with T1-T2 OPSCC undergoing surgery increased from 56 to 82% in the US, with a meta-analysis suggesting decreased toxicity associated with surgery compared to definitive CRT (Nichols et al., 2019). Although not a de-escalation trial, ORATOR evaluated QoL outcomes in primary RT/CRT vs. surgical intervention (Nichols et al., 2019) (Supplementary Table S1). In the surgical arm, 34 patients underwent TORS + neck dissection (ND), with 47% receiving adjuvant RT up to 64 Gy and 23.5% receiving CRT (RT + cisplatin 100 mg/m2 every 3 weeks). In the primary arm, 26.5% received RT up to 70 Gy and 67.6% received CRT. The primary outcome was powered to detect a 10-point difference in MDADI total mean scores at 1 year (higher is better). 1-year scores were 86.9 in the primary CRT arm vs. 80.1 in the surgical arm (*p* = 0.042). Grade 2 or higher adverse event rates (CTCAE) were similar in both arms, with preponderance for oral bleeding and trismus in the surgical arm and for neutropenia, hearing loss, tinnitus, and constipation in the primary CRT arm. TORS and ND were not associated with a superior QoL, and 3-year OS and PFS were 93 and 93.1%, respectively, with no differences between both arms (*p* = 0.89 and *p* = 0.63) (Nichols et al., 2019).

A follow-up prospective trial, ORATOR2, was planned to randomize patients to de-escalated CRT vs. de-escalating adjuvant treatment, the primary outcome being 2-year OS (Nichols et al., 2020). The results of a direct comparison between TORS and definitive CRT were eagerly awaited. Unfortunately, the trial was terminated due to unacceptable toxicity in the TORS + ND arm (two treatment-related deaths) (Palma et al., 2021), establishing primary CRT as a safe approach for treatment de-escalation. 61 patients were randomized in total. The 2-year OS was 100% for the RT arm vs. 83.5% in the TORS + ND arm (Palma et al., 2021). The findings of E3311, MC1765, and ORATOR are encouraging. Nonetheless, the data from ORATOR2 suggest that further studies will be needed to answer the question of surgery versus primary radiochemotherapy.

### 4.4 De-Intensification Schemes Using Immune Therapy

An emerging strategy is the combination of primary or adjuvant RT with modulators of the immune response, predominantly immune-checkpoint blockers (ICB, Supplementary Table S2).

HCC 18-034 (NCT03715946, 2018) is evaluating the addition of postoperative adjuvant reduced dose, moderately accelerated RT (45 or 50 Gy, in daily dose fx, six fx per week), and nivolumab (monoclonal antibody against Programmed cell Death protein 1 (PD-1)) in patients with advanced stage p16-IHC + OPSCC (seventh AJCC: T0, T3 + >2Nb and <10py or T0, T3 with >N1 and >10py) with intermediate risk features (ECE or positive margins). Nivolumab will be administered in two doses of 240 mg/m2 during weeks two and four of RT, and up to six doses afterward, of 480 mg/m2. The primary outcome is PFS at 3 years and gastrotomy tube dependence at 1 year (NCT03715946, 2018).

For definitive RT/CRT, NRG-HN005 is a prospective trial aiming to randomize 711 patients with p16-IHC + OPSCC, eighth AJCC stage I–II, and less than 10py to reduced dose RT (60 Gy in five fx, 6 weeks) with cisplatin, reduced dose RT (60 Gy in six fx, 5 weeks) with nivolumab, or standard of care (70 Gy RT in six fx, 5 weeks + cisplatin) (NCT03952585, 2019). The primary endpoint is 6-year PFS (NCT03952585, 2019).

NCT03799445 will evaluate the impact of upfront dual ICB with nivolumab and ipilimumab [monoclonal antibody against cytotoxic T-lymphocyte–associated protein 4 (CTLA4)], followed by RT (50–66 Gy) in patients with the eighth AJCC (stage I–II). In this trial, patients’ tumors must test positive for both p16-IHC and HPVDNA or RNA by ISH (NCT03799445, 2019). Primary endpoints include dose-limiting toxicity [DLT, defined as any ≥ grade III toxicity (CTCAE) related to immunotherapy not resolving within 28 days after treatment], complete response rate at 6 months, and 2-year PFS (NCT03799445, 2019).

The Canadian Cancers Trial Group CCTG HN.9 (NCT03410615) will randomize patients with p16-IHC + OPSCC and intermediate risk features (T1-2N1 smokers, T3N0-N1 smokers, and T1-3N2 any smoking history) in CRT 70 Gy/35 with cisplatin 100 mg/m^2^ or RT70Gy/35 with concurrent durvalumab 1500 mg (days 7 and 22), followed by durvalumab maintenance for six doses (Spreafico et al., 2018). The primary endpoint is event-free survival (EFS). A translational program including immunophenotyping, radiomic imaging, circulating tumor DNA (ctDNA), and microbiome analyses will be conducted in parallel (Spreafico et al., 2018).

The results of these trials are eagerly awaited, in light of the negative results from the Javelin Head and Neck 100 (**.**NCT02952586) and GORTEC 2017-01 (REACH, NCT02999087.) phase III trials. Javelin Head and Neck 100 compared the combination of avelumab, a PD-L1 inhibitor, + standard-of-care CRT (RT70Gy/2Gy + cisplatin 100 mg/m^2^ every 3 weeks) against standalone CRT in locally advanced HNSCC (Lee et al., 2021). The trial included HPV-positive and HPV-negative disease. The primary endpoint of PFS prolongation was not met, and there was no benefit seen upon stratification in HPV-positive disease (Lee et al., 2021). Similarly, in the GORTEC 2017-01-Reach trial, the combination of avelumab and cetuximab-based chemoradiotherapy did not improve PFS, further cementing the role of cisplatin-based radiochemotherapy as the standard of care in the treatment of locally advanced HNSCC (Bourhis et al., 2021).

Beyond ICB combinations, exploration of radiotherapy-induced immune activation and unmasking of HPV-associated neoepitope may, together with the growing arsenal of immunoncology (IO) drugs, facilitate the development of effective antitumor specific vaccines.

## 5 Molecular Stratification of HPV-Driven OPSCC

Beyond p16-IHC, direct HPV testing, and tobacco smoking, de-escalation trials can also contribute to a deeper understanding of the biology of HPV-driven OPSCC.

In one approach, PET imaging (18F-MISO PET detecting tumoral hypoxia) was used in a pilot study from the Memorial Sloan Kettering Cancer Center (MSKCC) to modulate the RT dose to the LNs in patients with p16-IHC + OPSCC receiving CRT (Lee et al., 2016). Patients with no baseline tumor hypoxia or with resolution of hypoxia after week 1 (per 18F-MISO PET scans) were candidates for 10-Gy dose de-escalation to the LNs (Lee et al., 2016). The primary tumor site received the standard RT dose (70 Gy). 10 patients (30%) were eligible for dose de-escalation (Lee et al., 2016). The 2-year clinical outcomes were as follows: 100% LC, 97% DM, and 100% for OS (Lee et al., 2016).

Additionally, targeting hypoxic HPV-driven tumors with heavy charged particles that are less dependent on the oxygen enhancement ratio (OER) (Klein et al., 2017; Chiblak et al., 2019) may provide another attractive venue to specifically escalate the dose while sparing normal tissue in this subgroup.

The association between the mutational landscape of HPV-driven OPSCC and patient outcomes is still under investigation. Beaty et al. performed next generation sequencing (NGS) of tumor samples from 78 patients enrolled in de-escalation trials of primary RT to investigate the prognostic role of PIK3CA mutations (Beaty B. T. et al., 2019). PIK3CA was the most significantly mutated gene in 21.8% of patients (Beaty B. T. et al., 2019; Beaty B. et al., 2019.). Patients with mutated PIK3CA had significantly lower 3-year DFS (65%) compared to patients with wild-type PIK3CA (93%, *p* = 0.0009), suggesting that this patient population is not suitable for de-escalation trials (Beaty B. T. et al., 2019; Beaty B. et al., 2019). However, conflicting data emerged from studies of patients with metastastic HPV-driven OPSCC where mutations in the PI3K pathway (PI3KCA, PIK3CA2B, and PIK3R1) were associated with an improved overall survival outcome at 5 years (Hanna et al., 2018).

Another biomarker trial approach is based on monitoring of circulating free DNA (cfDNA) or circulating tumor HPVDNA (ctHPVDNA) detected in patients’ blood. In NCT0316182, 115 patients were prospectively followed up for a median duration of 23 months after being treated with curative intent chemoradiotherapy (Chera et al., 2020). The trial estimated the positive predictive value (PPV) and negative predictive value (NPV) of ctHPV-DNA for determining disease recurrence (Chera et al., 2020). Undetectable levels of ctHPVDNA at post-treatment time points had an NPV of 100%. Conversely, two consecutively positive ctHPVDNA blood tests had a PPV of 94%. The median time from ctHPVDNA positivity to biopsy-proven recurrence was 3.9 months (Chera et al., 2020). cfHPV-DNA was confirmed as a highly specific biomarker of surveillance in a recent meta-analysis of 11 studies (Hanna et al., 2018). cfHPV-DNA had a pooled sensitivity of 0.81 (95% CI 0.78-0.84) and 0.98 (95%CI 0.96–0.99) at the first diagnosis. At follow-up, it had a sensitivity of 0.73 (95%CI 0.57–0.86) and a specificity of 1 (Hanna et al., 2018). Interestingly, one study found a significant association between levels of cfHPV-DNA and N status, as well as the extent of disease involvement (Tanaka et al., 2022). Levels of cfHPV-DNA increased as function of involvement, with the lowest in locally advanced disease, followed by locoregional spread, and the highest for distant metastases (Tanaka et al., 2022).

Similarly, HPVDNA may be detected from oral rinses. Patients with persistent oral HPVDNA after the end of therapy had a decreased 2-year OS (HR = 1.86, *p* = 0.003) compared to patients without detectable DNA in a prospective phase II clinical trial (Fakhry et al., 2019). Finally, the association between seropositivity to HPV16 antigens and clinical outcomes has been demonstrated in several studies (Dahlstrom et al., 2015; Nelson et al., 2017). Furthermore, a recent study investigated differential patterns of antibody response to cancer antigens in HPV-driven versus HPV-negative HNSCC: antibodies against IMP-1 (found in *n* = 9/153, 6% of patients) were adversely prognostic only in HPV-driven OPSCC (HR = 3.28, *p* < 0.001) (Laban et al., 2019). Detecting relevant tumor immune microenvironment (TIME) parameters may also assist in stratifying risk for recurrence and inferior OS in HPV-driven HNSCC. A multicentric retrospective study from the German Cancer Consortium (DKTK) identified enrichment in CD8^+^ infiltrating immune cells as an independent prognostic factor both in p16-IHC+/HPVDNA + OPSCC tumors and HPV-negative tumors, in a cohort of 161 patients treated with surgery and postoperative CRT (Balermpas et al., 2016). Disappointingly, high levels of PD-L1 were not prognostic in the Javelin 100 Head and Neck trial, although this finding was not stratified by HPV status (Lee et al., 2021).

Taken together, these findings reaffirm the importance of validating all biomarkers in prospective phase III clinical trials.

## 6 Conclusion

The identification of HPV as a protoypic predictive marker for molecular stratification of patients has paved the way for the development of several avenues of treatment de-intensification. Individualized therapy may be tailored by de-escalation or adaption of local radiotherapy and de-intensification/replacement of systemic therapy. This field is evolving at a rapid pace. In this dynamic era, deeper understanding of the biology of HPV-driven tumors shapes physicians’ approach toward improved diagnosis, staging, risk stratification, and management of this disease. With a steadily increasing complexity and a plethora of opportunities, a consensus is needed to assure better comparability, for example, homogenizing inclusion criteria, exclusion criteria, risk stratification (most noteworthy, the impact of smoking), staging, and diagnosis of an HPV-driven tumor, replacing the single p16-immunohistochemistry test with a combination of direct HPV tests or including more advanced molecular methods that better assist in stratifying patients at low risk for locoregional or distant recurrence.

In postoperative adjuvant treatment of OPSCC, the design of current trials accounts for extracapsular extension, although it was not included as an adverse prognosis factor in the eighth AJCC staging system. In primary definitive CRT treatment, data from phase III trials, where substitution of cisplatin with cetuximab leads to inferior survival outcomes, have established cisplatin-based radiochemotherapy as the standard of care. Similarly, results from NRG-HN002 consolidated the role of cisplatin, whereby a higher rate of locoregional recurrence was found in patients treated with moderately accelerated radiotherapy alone (60 Gy in 5 weeks) and complete omission of cisplatin. Nevertheless, numerous other strategies are ongoing, with promising data emerging from phase II clinical trials. Confirmation in randomized phase III clinical trials is awaited. Maturity of follow-up will be an issue to address in these trials, given the main pattern of distant relapse after 2 years in HPV-driven OPSCC.

Multiparametric tumor characterization may be needed for accurate patient selection to avoid de-escalating patients at high risk of recurrence. In parallel, broadening the therapeutic window with targeted tumor-specific agents, a growing immunoncology arsenal, and novel radiation dose/quality painting *via* heavy charged particles may navigate the therapy of HPV-driven HNSCC toward high-precision oncology.
