# Supplementary material for: Evolution of a Paradigm Switch in Diagnosis and Treatment of HPV-Driven Head and Neck Cancer—Striking the Balance Between Toxicity and Cure
Source: Front Pharmacol. 2022 Jan 20;12:753387. doi: 10.3389/fphar.2021.753387 (PMC8810823; doi:10.3389/fphar.2021.753387)
Supplement: Supplementary file 1 [file DataSheet1.docx]

Supplementary Table 1. Selection of de-escalation trials

| **Study Name ID** | **7^th^ AJCC, Smoking** | **8^th^ AJCC equivalent** | **Design** | **HPV testing** | **Primary Endpoint** |
| --- | --- | --- | --- | --- | --- |
| *Surgery and modulation of adjuvant radiochemotherapy* | | | | | |
| ADEPT  NCT01687413  N=41/500 | T1T4a-N+ M0, R0, ECE+ | I,II,III | TORS then  Arm A: 60Gy IMRT  Arm B: 60 Gy IMRT + cisplatin (40mg/m2) | p16-IHC | 2yr DFS  2yr LC  Terminated due to slow accrual |
| PATHOS  NCT02215265^13^  N=242 (phase II)  N=1100 (Phase III) | T1T3-N0N2b  Excludes active smokers with N2b | I,II | TORS then  Low risk:   - Arm A: TORS   Intermediate risk: T3, pN2a-N2b, LVI, pNI, close margin   - Arm B1: TORS + 50Gy - Arm B2: TORS + 60Gy   High risk:   - R1: RT+ 60 Gy - ECE+: RT + 60Gy + cisplatin (100mg/m^2^) | p16 IHC and HPVDNA ISH | Phase II: Co-primary endpoint 1yr MDADI and OS  Phase III: 5-year OS (non-inferiority) |

Supplementary Table 2. Selection of ongoing de-escalation trials with immunotherapy

| **Study Name ID** | **7^th^ AJCC, Smoking** | **8^th^ AJCC equivalent** | **Design** | **HPV testing** | **Primary Endpoint** |
| --- | --- | --- | --- | --- | --- |
| *De-escalation trials with immunotherapy* | | | | | |
| NRG-HN005  NCT03952585  N=711 |  | T1T2-N1  T3-N0N1  <10 py | Arm I: 70Gy RT + cisplatin  Arm II: 60Gy + cisplatin  Arm III: 60Gy RT + nivolumab | p16-IHC | Phase II:  PFS (non-inferiority)  Phase III: co-primary endpoints:  PFS (6yr, non-inferiority)  superiority of QoL (MDADI score) |
| **NCT03799445**  **N=180** | T1-N2aN2c  T2-N1N2c  T3-N0N2c | I, II | 2 cycles Nivolumab + Ipilimumab then 50-66Gy RT | p16-IHC and HPVDNA ISH or HPVRNA ISH (RNAscope) | Acute Dose Limiting toxicity (DLT measured by CTCAE v4)  6 months Rate of Complete Response 2yr PFS |
| NCT02764593  N=40  RTOG 3504 | Oral cavity, Larynx, hypopharynx, Oropharynx  OPSCC:  >10 py, T1T2-N2bN3 or  <10 py + T4N0N3 or T1T3N3 | III | Arm 1: Nivolumab then 70Gy RT + reduced dose cisplatin then Nivolumab  Arm 2: Nivolumb then 70Gy RT + cisplatin 100mg/m2 then Nivolumab  Arm 3: Nivolumab then 70Gy RT + Cetuximab then Nivolumab  Arm 4: Nivolumab then 70Gy RT then Nivolumab | p16-IHC | DLT (CTCAE, v4) |
| HCC 18-034  NCT03715946  N=135 | Intermediate risk:  If <10 py: T0T3 + >N2b, N2c/N3 or ECE+ or R1  If >10 py: T0T3 + N2N3 or ECE+ or R1 | II,III | TORS + RT (45-50Gy) + Nivolumab | p16-IHC | 3yr PFS  1yr PEG tube dependence |
